# Associations between the atherogenic index of plasma and psoriasis among US adults: A cross-sectional study based on NHANES 2009 to 2014

**DOI:** 10.1097/MD.0000000000040955

**Published:** 2024-12-13

**Authors:** Yanan Tuo, Junchen He, Tao Guo

**Affiliations:** a Department of Dermatology, Tianjin Academy of Traditional Chinese Medicine Affiliated Hospital, Tianjin, China.

**Keywords:** NHANES, psoriasis, the atherogenic index of plasma

## Abstract

The atherogenic index of plasma (AIP) is a significant indicator of lipid levels. This study aimed to investigate the association between psoriasis and AIP in adults. The association between AIP and psoriasis was investigated using multivariate logistic regression, and smoothing curve fitting utilizing data from the National Health and Nutrition Examination Survey 2009 to 2014. Subgroup analysis and interaction tests were employed to investigate whether this relationship was stable across populations. The final sample included 8177 participants, representing approximately 60 million people in the US. Psoriasis among the AIP groups (quartile, Q1–Q4) was statistically significant (*P* < .05). In the minimally adjusted model, each 1-unit increase in AIP was associated with a 44% increase in the risk of developing psoriasis [1.44 (1.01, 2.20)]. Participants in the highest quartile of AIP had a 40% higher risk of developing psoriasis than those in the lowest quartile [1.40 (1.05, 2.10)]. In the male group, the risk of developing psoriasis increased by 0.86 points per 1 unit increase in AIP. AIP is positively associated with psoriasis in US adults. Our findings imply that AIP improves psoriasis prevention in the general population.

## 1. Introduction

Psoriasis is a relapsing, chronic dermatosis that affects about 3% of individuals in the US.^[1]^ Its prevalence has been steadily rising.^[2,3]^ Recently, psoriasis has now recognized as a multi-organ system inflammatory disease that can cause metabolic syndrome, inflammatory bowel disease, diabetes, and cardiovascular disease.^[4,5]^ Systemic inflammation caused by metabolic or immunological conditions is strongly linked to psoriasis.

The atherogenic index of plasma (AIP) was first proposed by Dobiásová as a biomarker for plasma atherosclerosis.^[6]^ AIP, a novel index made up of triglyceride (TG) and high-density lipoprotein cholesterol (HDL-C) levels, is computed using the formula log (TG/HDL-C).^[7]^ AIP is a powerful predictor of atherosclerosis and coronary heart disease in addition to accurately illustrating the real link between protective and atherogenic lipoproteins.^[8]^ Furthermore, in addition to conventional risk factors, the AIP provides an independent prognostic marker for fast plaque progression^[9]^ and might be an effective biomarker that could be used to predict the risk of cardiovascular events among patients with psoriasis.

There appears to be a significant overlap between the inflammatory responses underlying psoriasis and atherosclerosis lesions. It has been suggested that aberrant lipid metabolism may cause elevated atherosclerosis risk in psoriatic patients.^[10]^ It has been demonstrated that serum lipids influence the severity of psoriasis, with lipid-mediated inflammatory reactions being a major factor.^[11]^ Moreover, a cross-sectional study including thirty-nine patients has shown that AIP is a predictor of psoriatic arthritis.^[12]^ However, no research has been done to investigate the connection between AIP and psoriasis. In fact, the number of patients with psoriasis is much larger than the number of patients with psoriatic arthritis. This cross-sectional investigation was carried out using data from the 2009-2014 the National Health and Nutrition Examination Survey (NHANES).

## 2. Materials and methods

### 2.1. Study population

**Figure 1.**
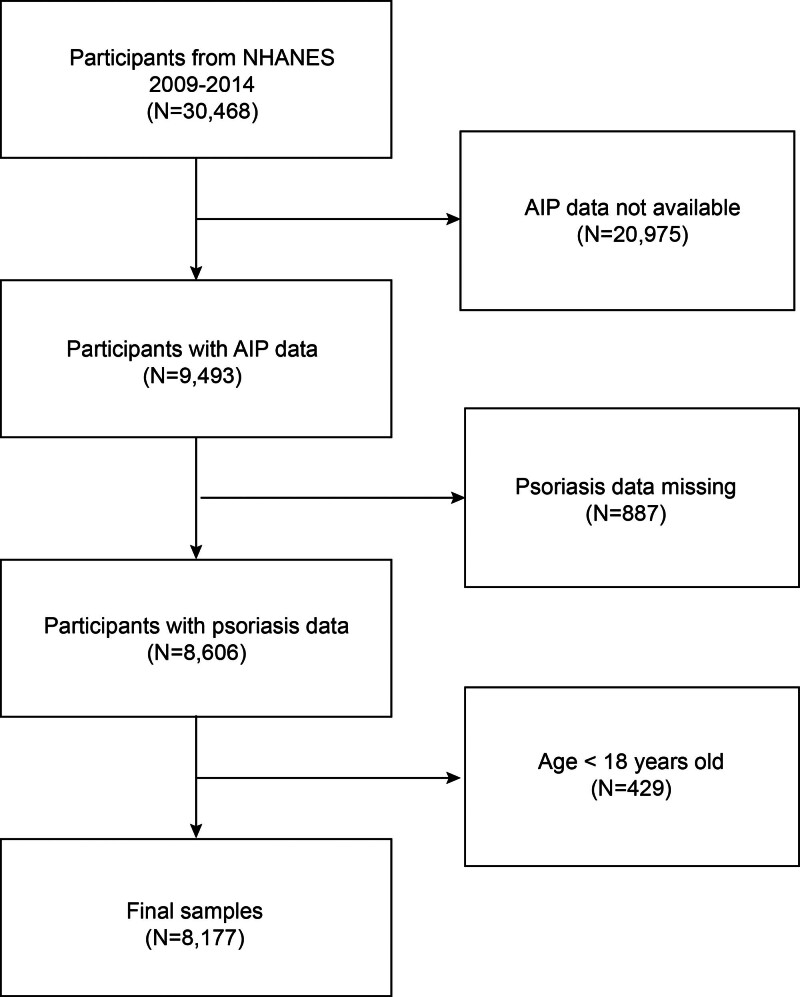
Flow chart of participants selection. NHANES = National Health and Nutrition Examination Survey.

The NHANES is a continuous survey designed to assess the health and nutritional condition of American adults and children. NHANES uses a complex, multistage, probability sampling design (a complex, multistage, probability sampling design) with 4 stages: counties, urban neighborhoods, households, and individuals). This sampling design ensures a representative and broad sample.^[13,14]^ The Ethics Review Committee of the National Center for Health Statistics gave its approval to the NHANES research plan. Each research participant provided written informed consent.^[15,16]^ More comprehensive information can be found at www.cdc.gov/nchs/nhanes/irba98.htm. The survey was conducted during 3 survey cycles over 6 years (2009–2014). Psoriasis data were only available to individuals aged 16 to 80 in the NHANES 2009 to 2014 cycles. We excluded 887 participants without available psoriasis data, 20,975 participants with missing or incomplete AIP data, and 429 participants under 18 years old. The study eventually included 8177 participants (Fig. 1).

### 2.2. Atherogenic index of plasma

The AIP was the exposure variable. It was defined as log10 (TG/HDL-C), where mmol/L is the unit of measurement for both TG and HDL-C.^[17]^

### 2.3. Diagnosis of psoriasis

In response to the question, “Have you ever been told by a health care provider that you had psoriasis?” psoriasis was self-reported.

### 2.4. Covariables

Covariates included age, race, gender, smoking status, alcohol drinking status, coronary heart disease, high blood pressure, diabetes, low-density lipoprotein cholesterol (LDL-C), albumin, income-to-poverty ratio, education level, body mass index (BMI) and waist circumference.

### 2.5. Statistical analysis

The participant’s demographics were evaluated by AIP quartile utilizing the chi-square and t-tests. The linear relationships between AIP and psoriasis were examined using weighted multivariate linear and logistic regression models. After converting the AIP score from a continuous to a categorical variable (quartile), a trend test was performed to investigate the trend of linear connection between AIP and psoriasis. Subgroup analysis was used to investigate the association between AIP and psoriasis in US adults of different age, gender, BMI, and diabetes status, and interaction tests were used to investigate whether the relationship were consistent across subgroups. The nonlinear association between AIP and psoriasis was examined using smoothing curve fitting.^[18,19]^ R (version 4.2) or Empowerstats (version 5.0) were used for all analyses. The definition of statistical significance is 2-sided *P* < .05.

## 3. Results

### 3.1. Baseline characteristics

Of a total of 8177 participants older than 18 years, the mean (SD) age was 47.82 (18.48) years, with 51.73% female and 43.10% non-Hispanic White. A total of 213 participants (2.60%) had psoriasis, and 7964 (97.40%) did not have psoriasis. The mean (SD) AIP for all participants was 0.3063 (0.3267), with an interquartile range of quartile 1: <0.072; quartile 2: 0.073 to 0.288; quartile 3: 0.289 to 0.508; quartile 4: >0.509. Participants with a higher AIP were more likely to be older, male, and non-Hispanic White, higher level of education and to have a higher likelihood of diabetes, coronary heart disease, high blood pressure, smokers, arthritis, chronic bronchitis and psoriasis compared with the lowest AIP quartile. Furthermore, participants with higher AIP typically had higher BMI, waist circumference, LDL-C, and less statin use (Table 1).

**Table 1:** Basic participant characteristics based on the atherogenic index of plasma among US adults.

Characteristics	Atherogenic index of plasma				*P* value
	Q1 (N = 2023)	Q2 (N = 2064)	Q3 (N = 2042)	Q4 (N = 2048)	
Age (yr)	44.45 ± 18.89	47.58 ± 19.13	49.81 ± 18.44	49.40 ± 16.91	<.001
Gender (%)					<.001
Male	34.60	45.54	50.93	61.87	
Female	65.40	54.46	49.07	39.13	
Race/ethnicity (%)					<.001
Mexican American	9.39	13.91	16.75	19.29	
Other Hispanic	8.01	10.13	10.77	12.35	
Non-Hispanic White	39.50	42.39	42.46	48.05	
Non-Hispanic Black	30.00	21.27	17.53	10.21	
Other races	13.10	12.31	12.49	10.11	
Education level (%)					<.001
<High school	17.61	23.48	27.44	31.86	
High school	18.74	21.88	22.84	23.10	
>high school	63.65	54.64	49.72	45.05	
Smoked at least 100 cigarettes in life (%)					<.001
Yes	35.55	40.15	44.59	51.99	
No	64.45	59.85	55.41	48.01	
Diabetes (%)					<.001
Yes	5.29	9.01	13.81	17.77	
No	94.71	90.99	86.19	82.23	
Coronary heart disease (%)					<.001
Yes	2.33	3.45	4.70	5.08	
No	97.67	96.55	95.30	94.92	
High blood pressure (%)					<.001
Yes	26.15	32.85	38.34	43.60	
No	73.85	67.15	61.66	56.40	
Family PIR	2.57 ± 1.69	2.47 ± 1.66	2.36 ± 1.60	2.18 ± 1.57	<.001
BMI	25.95 ± 6.32	28.13 ± 6.80	29.80 ± 6.90	31.37 ± 6.62	<.001
Waist circumference (cm)	89.38 ± 14.61	96.33 ± 15.75	101.03 ± 15.53	106.11 ± 15.22	<.001
LDL-C (mg/dL)	101.58 ± 30.15	112.03 ± 33.24	117.70 ± 36.17	119.21 ± 38.02	<.001
Albumin (g/dL)	4.27 ± 0.32	4.25 ± 0.34	4.21 ± 0.33	4.23 ± 0.35	<.001
How often have you drunk alcohol over the past 12 mo	4.70 ± 21.07	4.17 ± 22.76	4.88 ± 38.56	4.84 ± 37.68	.917
Psoriasis (%)					.006
Yes	2.03	2.28	3.13	3.22	
No	97.97	97.72	96.87	96.78	
Asthma (%)					.411
Yes	14.82	13.28	13.77	15.48	
No	85.18	86.72	86.23	84.52	
Arthritis (%)					<.001
Yes	22.14	24.57	27.80	28.49	
No	77.86	75.43	72.2	71.51	
Thyroid problems (%)					.926
Yes	10.08	9.76	10.57	9.30	
No	89.92	90.24	89.43	90.7	
Chronic bronchitis (%)					<.001
Yes	4.36	3.74	5.82	6.67	
No	95.64	96.26	94.18	93.33	
Statin use (%)					.004
Yes	82.87	84.16	81.58	74.47	
No	17.13	15.84	18.42	25.53	

Mean ± SD for continuous variables: the *P* value was calculated by the weighted linear regression model; (%) for categorical variables: the *P* value was calculated by the weighted chi-square test.

BMI = body mass index, LDL-C = low-density lipoprotein, PIR = the ratio of income to poverty.

### 3.2. Association between AIP and psoriasis

**Figure 2.**
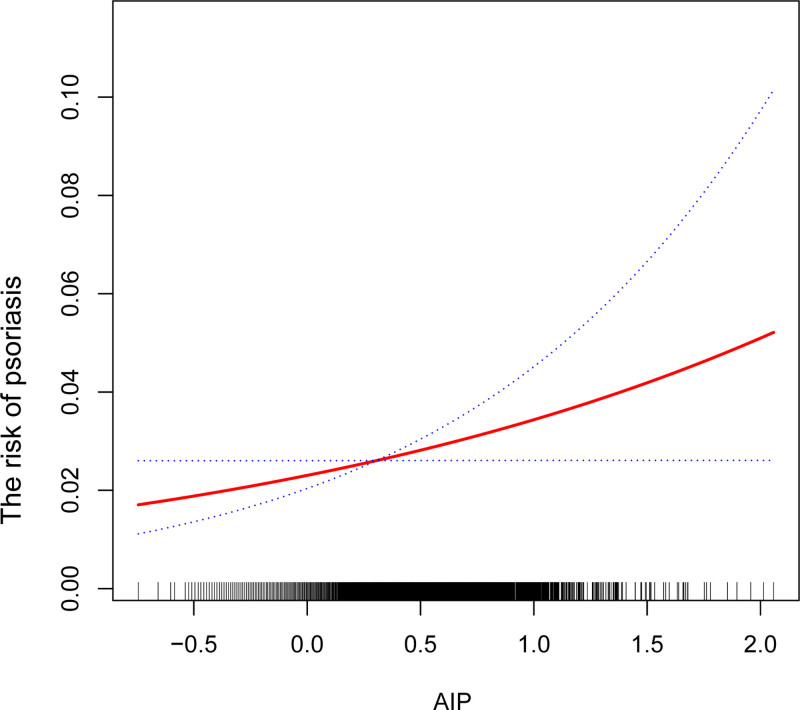
The nonlinear associations between AIP and psoriasis. The solid red line represents the smooth curve fit between variables. Blue bands represent the 95% of confidence interval from the fit. AIP = atherogenic index of plasma.

Table 2 shows the associations between AIP and psoriasis. The results showed a significant positive association between AIP and psoriasis in both the crude model [1.57 (1.05, 2.35)] and minimally adjusted model [1.44 (1.01, 2.20)], but this association became insignificant in the fully adjusted model [0.98 (0.56, 1.85)]. After AIP was categorized as quartiles, the above connection remained statistically significant (*P* for trend < .01). Participants in the highest quartile of AIP had a 40% increased risk of developing psoriasis [1.40 (1.05, 2.10)] compared to participants in the lowest quartile of AIP in minimally adjusted model. The smoothed curve fitting results provided additional support for the nonlinear positive correlation between AIP and psoriasis (Fig. 2).

**Table 2:** Association between the atherogenic index of plasma and psoriasis.

AIP	Psoriasis	*P* for trend
	OR (95% CI)	
Crude model (Model 1)		
Continuous	1.57 (1.05, 2.35)	
Categories		.0232
Quartile 1	0 (ref)	
Quartile 2	1.13 (0.74, 1.8772)	
Quartile 3	1.56 (1.05, 2.33)	
Quartile 4	1.62 (1.02, 2.27)	
Minimally adjusted model (Model 2)		
Continuous	1.44 (1.01, 2.20)	
Categories		.0211
Quartile 1	0 (ref)	
Quartile 2	1.08 (0.71, 1.72)	
Quartile 3	1.37 (0.98, 2.18)	
Quartile 4	1.40 (1.05, 2.10)	
Fully adjusted model (Model 3)		
Continuous	0.98 (0.56, 1.85)	
Categories		.6755
Quartile 1	0 (ref)	
Quartile 2	0.92 (0.69,1.42)	
Quartile 3	0.83 (0.67, 1.27)	
Quartile 4	1.25 (0.91, 1.33)	

Model 1: no covariates were adjusted. Model 2: age, gender, and race were adjusted. Model 3: age, gender, race, education level, PIR, BMI, drinking alcohol, smoking, diabetes, coronary heart disease, high blood pressure, waist circumference, LDL-C, asthma, arthritis, thyroid problems, chronic bronchitis, statin use and albumin were adjusted.

AIP = atherogenic index of plasma, BMI = body mass index, LDL-C = low-density lipoprotein, PIR = the ratio of income to poverty.

### 3.3. Subgroup analyses

We conducted interaction tests and subgroup analysis, stratified by age, gender, BMI, and diabetes, to identify potential distinct demographic settings and ascertain if the relationship between AIP and psoriasis was consistent in the general population (Table 3). Our results showed that the association between AIP and psoriasis was significantly different between gender subgroups (*P* for interaction < .05). In the male group, the risk of developing psoriasis increased by 0.86 points per 1 unit increase in AIP. In contrast, among those female, the association between AIP and psoriasis became a non-significant positive association.

**Table 3:** Subgroup analysis of the association between AIP and psoriasis.

Subgroup	Psoriasis (OR [95%CI])	*P* for interaction
Sex		.0423
Male	1.86 (1.12, 2.55)	
Female	1.39 (0.72, 1.92)	
Age (yr)		.5452
<60	1.43 (0.77, 2.12)	
≥60	1.80 (0.94, 3.98)	
BMI (kg/m^2^)		.7691
<24.9	1.55 (0.65, 3.89)	
25–29.9	1.05 (0.43, 3.12)	
≥30	1.25 (0.76, 2.57)	
Diabetes (%)		.9813
Yes	1.41 (0.62, 3.89)	
No	1.58 (0.85, 2.17)	

Age, gender, race, education level, PIR, BMI, drinking alcohol, smoking, diabetes, coronary heart disease, high blood pressure, waist circumference, total cholesterol, HDL-C, asthma, arthritis, thyroid problems, chronic bronchitis, statin use and albumin were adjusted.

BMI = body mass index, HDL-C = high-density lipoprotein cholesterol, PIR = the ratio of income to poverty.

## 4. Discussion

The cross-sectional study, which included 8177 representative adults, showed positive correlations between AIP and psoriasis, suggesting that dyslipidemia could be associated with a higher risk of psoriasis development. The AIP can be an effective method for identifying individuals at high risk of psoriasis. Our findings imply that AIP might be useful clinically for determining the severity and risk of psoriasis.

To our knowledge, few research assess the relationship between AIP and psoriasis. Aksoy et al conducted a cohort study among 142 individuals to assess the relationship between AIP levels and psoriasis. The results showed that in patients with psoriasis, AIP (0.10 ± 0.24 vs −0.04 ± 0.27, *P* = .001) were significantly higher in patients with psoriasis.^[20]^ Sunitha et al^[21]^ reported that AIP was significantly higher in patients with psoriasis than in healthy controls and positively correlated with psoriasis area severity index. Similarly, researches have shown that individuals with psoriasis have decreased HDL-C levels but increased triglyceride, cholesterol, and LDL-C levels when compared to the control group.^[22–24]^ In another meta-analysis, assessing the connection between psoriasis and metabolic syndrome, the rate of metabolic syndrome was statistically significant 2.07 times higher in patients with psoriasis than in control groups, and it was recommended that psoriasis patients should be clinically monitored closely for high blood pressure, elevated fasting blood sugar, elevated triglycerides, and low HDL-C, which are linked to the metabolic condition.^[25]^ In our study, we evaluated AIP as a risk factor for cardiovascular diseases, patients with psoriasis had significantly higher AIP when adjusted for major demographic variables. There are research that support our findings by showing that psoriasis patients have higher atherogenic index levels.^[21,26]^ According to our study’s results, which are in line with previous research, those who have high levels of the atherogenic index when they are dealing with psoriasis run a higher risk of experiencing cardiovascular problems. We further evaluated the relationship between the AIP and psoriasis by sex and found that there was a positive correlation between the AIP and outcome events among males but not among females. Significant sex differences were discovered in the cross-sectional study, which may have been caused by the participants’ varied characteristics. Moreover, there are also epidemiological studies show that the risk of psoriasis is higher among males in some locations of lesions.^[27,28]^ McDonald and Calabresi’^[29]^ suggested that patients with psoriasis are more likely to experience thromboembolic events and atherosclerotic cardiovascular disease, especially in men.

However, a cross-sectional study from Iran showed that there was no significant difference in lipid levels between patients with psoriasis and the control group.^[30]^ In the study by Uyanik, while TG levels were considerably greater in psoriasis patients compared to controls, TC and LDL-C levels were not significantly higher. Additionally, no patient’s HDL-C values were statistically significantly low (*P* > .05).^[31]^ In other large clinical observational studies, no clear connection has been shown between the type of dyslipidemia and psoriasis. Kaur et al suggested the TG level may be the mechanism behind the psoriasis. Nonetheless, the pathophysiological link between TG and psoriasis remained vague as a result of a lack of pertinent research.^[32]^

Psoriasis is a chronic systemic inflammatory disease characterized by keratinocyte anomalies and immune dysfunctions accompanied by various comorbidities.^[33]^ The pathophysiology of psoriasis may also be significantly influenced by abnormalities in lipid metabolism. Changes in plasma lipid and lipoprotein levels, elevated oxidative stress, and reduced antioxidant capacity make psoriasis patients more vulnerable to cardiovascular complications including myocardial infarction or stroke, as well as atherogenicity.^[34]^ It is believed that persistent inflammation or treatment-related dyslipidemia raises the pro-atherogenic lipid profile tendency in psoriasis patients, increasing their risk of cardiovascular events and dyslipidemia-related diseases.^[35,36]^ Pro-inflammatory cytokines, like tumor necrosis factor-a, interleukin 6, and interferon gamma, increase lipoprotein lipase activity in the endothelium wall after chronic inflammation, which raises LDL-C levels and, as a result, a dyslipidemic state that raises the risk of cardiovascular disease.^[23]^ AIP is linked to the size of pre- and anti-atherogenic lipoprotein particles and reflects the genuine relationship between protective and atherogenic lipoprotein.^[37]^ Since both cardiovascular disease and psoriasis are inflammatory conditions mediated by T helper 1/T helper 17, psoriasis may be regarded as a personal risk factor for the development of cardiovascular events.

One of our study’s strengths is our intricate multi-stage probability sampling design, which improved the study’s representativeness and dependability. Our study has several limitations. First, this was a cross-sectional study, a design that is less thorough than a cohort study. In addition, this study’s capacity to test the etiology hypothesis and extrapolation was insufficient, and its ability to investigate the etiology hypothesis was constrained. Other cohort studies are needed to verify the correlation between the AIP and psoriasis. Secondly, the possibility of confusion due to unidentified or immeasurable causes cannot be totally ruled out. Reliance on self-reported psoriasis status may introduce reporting bias and lack of clinical validation. In addition, the data on psoriasis patients is low and the severity of the disease is not delineated in detail, which may have affected the results of the study.

## 5. Conclusion

This cross-sectional study was conducted among 8177 American adults and showed a positive correlation between the AIP and psoriasis, and the risk of psoriasis increased gradually with the increase in the AIP. Moreover, we found an interaction between sex and the AIP and found that the AIP was positively correlated with psoriasis only among men and not among women. The AIP may be of potential value in clinical practice for identifying psoriasis severity. More excellent prospective investigations need to confirm our conclusions regarding this research topic.

## Acknowledgments

We want to thank all participants in this study.

## Author contributions

**Conceptualization:** Yanan Tuo.

**Data curation:** Yanan Tuo.

**Formal analysis:** Yanan Tuo.

**Investigation:** Junchen He.

**Methodology:** Yanan Tuo, Junchen He.

**Project administration:** Junchen He.

**Resources:** Tao Guo.

**Software:** Tao Guo.

**Writing – original draft:** Yanan Tuo.

**Writing – review & editing:** Tao Guo.
